# Highly Stable Pt-Based Oxygen Reduction Electrocatalysts toward Practical Fuel Cells: Progress and Perspectives

**DOI:** 10.3390/ma16072590

**Published:** 2023-03-24

**Authors:** Miao-Ying Chen, Yuan Li, Hao-Ran Wu, Bang-An Lu, Jia-Nan Zhang

**Affiliations:** College of Materials Science and Engineering, Zhengzhou University, Zhengzhou 450001, China

**Keywords:** oxygen reduction reaction, platinum, durability, fuel cells, intermetallic structure

## Abstract

The high cost and poor reliability of cathodic electrocatalysts for the oxygen reduction reaction (ORR), which requires significant amounts of expensive and scarce platinum, obstructs the broad applications of proton exchange membrane fuel cells (PEMFCs). The principles of ORR and the reasons for the poor stability of Pt-based catalysts are reviewed. Moreover, this paper discusses and categorizes the strategies for enhancing the stability of Pt-based catalysts in fuel cells. More importantly, it highlights the recent progress of Pt-based stability toward ORR, including surface-doping, intermetallic structures, 1D/2D structures, rational design of support, etc. Finally, for atomic-level in-depth information on ORR catalysts in fuel cells, potential perspectives are suggested, such as large-scale preparation, advanced interpretation techniques, and advanced simulation. This review aims to provide valuable insights into the fundamental science and technical engineering for practical Pt-based ORR electrocatalysts in fuel cells.

## 1. Introduction

In the past few decades, the booming development of nanotechnology has driven the continuous progress and development of nanocatalysis science and technology, leading to the emergence of nanocatalysis. Nanocatalysts exhibit unique physicochemical properties at the nanoscale, such as size effects [1], surface effects [2], synergistic effects [3], etc., making metal nanocatalysts exhibit excellent performance in many reactions, and they have important applications in energy conversion, waste gas treatment, drug preparation, chemical production, and other fields. The energy problem is crucial to human development, and we have a responsibility and obligation to develop green and sustainable new energy. As a clean and efficient secondary energy source, hydrogen energy can play a big part in addressing the problem of global climate change [4,5,6]. Due to their high energy density, low operating temperature, environmental friendliness, independence from the Carnot cycle, and hydrogen energy recycling and usage, PEMFCs are considered the most promising sustainable energy storage and conversion device [7,8,9,10]. However, their performance, cost, and durability still limit large-scale PEMFC commercialization [11,12,13,14].

The slow kinetics of the cathodic ORR, which requires significant amounts of expensive and scarce platinum, obstructs the large-scale use of PEMFCs [15,16,17]. There have been considerable advances in the mechanistic understanding of ORR, the development of new catalyst manufacturing techniques, and overall performance enhancements over the past few decades [18,19]. Numerous catalysts, including Pt alloys, Pd alloys, metal chalcogenides, non-noble metal-based materials, etc., have been synthesized and studied [20,21,22,23,24,25]. Although non-noble metal and nitrogen co-doped carbon materials have attracted considerable research interest and have been regarded as the most promising catalysts to replace Pt-based catalysts for ORR, especially typical Fe-N-C, their poor durability under acidic conditions hinders their practical application in PEMFCs [26,27,28,29]. Therefore, it is of great importance to research Pt-based electrocatalysts with highly efficient activity and stability for ORR. Inspired by the structure effects established by model surfaces and theoretical results, it has been recognized that precise control over nanoscale Pt structures, along with their morphology and composition control/regulation, is the most effective way to intrinsically improve ORR performance and reduce the amount of expensive-but-rare Pt required [4,7,30]. For the performance optimization of Pt-based nanocatalysts, Pt alloy catalysts are undoubtedly a good choice. Markovic et al. [31] have shown that the Pt_3_Ni (111) surface is ten times more active toward ORR than the analogous Pt (111) surface and that this owes to the Pt_3_Ni surface’s remarkable electronic structure and surface atom arrangement at the near-surface area. Pt_3_Ni (111) is 90 times more active than Pt/C catalysts, which are state-of-the-art catalysts for PEMFCs. By analyzing the degree of dealloying following electrochemical cycling, Sun and coworkers [32] demonstrated that the stability of square-ordered FePt nanoparticles is substantially higher than that of face-centered cubic FePt nanoparticles. Surface modification is also a powerful technique. By increasing the oxidation potential of active sites on the surface of nano-Pt with gold cluster modification, Adzic et al. [33] were able to reduce Pt dissolution and migration. Hence, Pt nanoparticles (NPs) show negligible variation in their electrochemical active surface area throughout cycling tests, whereas Pt/C without Au modification exhibited a severe decrease in activity catalysts. In addition to the above methods, some of the most effective improvement measures are the doping of metal atoms, which promotes surface restructuring; the synthesis of self-supporting materials; and the optimization of carbon supports.

After 5000 h of operation (equal to 250,000 km) and thousands of start-up and shut-down events, a fuel cell should retain at least 90% of its performance to compete with traditional internal combustion engines [34]. Because of the intense Ostwald ripening, Pt NPs readily detach from carbon supports and aggregate into big NPs, causing substantial stability difficulties for these catalysts during fuel cell operation. Several studies have found that carbon support corrosion and metal dissolution/aggregation are the primary causes of catalyst deterioration [35]. Optimizing the performance of PEMFCs requires a systematic approach to build catalysts from metals [12,36] with optimal activity and durability and from supports [22,37] with enhanced stabilities. In brief, the use of stable and efficient Pt-based ORR catalysts in fuel cells and other comprehensive energy technologies is an area of particular interest.

Herein, we provide a concise summary of the fundamentals of ORR and the mechanisms of degradation for Pt-based catalysts during practical applications, followed by a discussion of the current methods of stabilizing Pt-based catalysts in processes associated with PEMFCs: optimizing the metal’s structure to increase its stability, i.e., intermetallic compound structure; the use of dimensional effects; the introduction of additional metal elements; and the advancement of support materials, including graphite, N-doped carbon, and composite supports. Atomic-level research on ORR catalysts for fuel cells can point the way toward future advancements in areas such as mass production, better understanding, and more accurate simulation. This review systematically summarizes the existing work on improving the stability of Pt-based catalysts and classifies and summarizes these stability improvement strategies in detail, and we hope this review will serve as a call to action for deeper investigation into unique research methodologies in this area.

## 2. The Fundamentals of ORR

**Figure 1:**
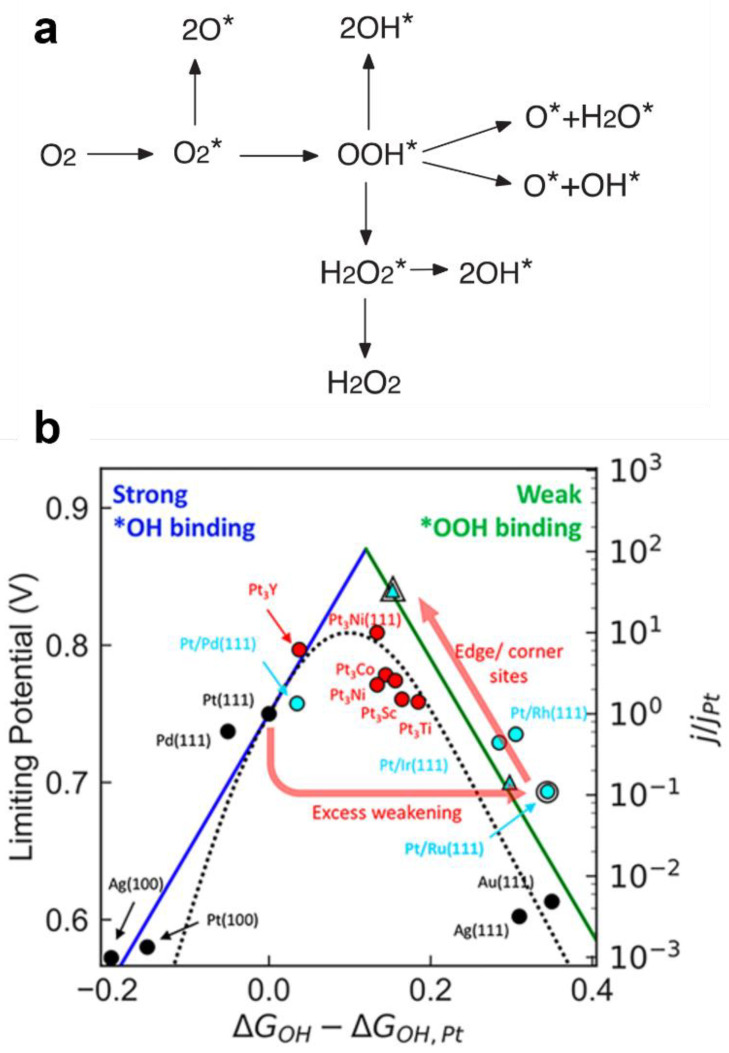
(**a**) Schematic illustration of the possible ORR pathways: initial reduction and decomposition of O_2_. Species with an asterisk (*) are adsorbed. Reprinted with permission from [40]. Copyright 2012, IOP Publishing. (**b**) Comparison between dynamic volcanoes (based on micromechanics modeling) and extreme potential volcanoes (based on thermodynamic analysis) shows that both approaches have successfully predicted the observed activity trend of a series of materials. Red circles, cyan circles, highlighted cyan circle, and highlighted cyan triangle represent Pt_3_M alloys (M = Co, Ni, etc.), M-loaded Pt species (M = Ru, Rh, etc.), Ru@Pt, and Ru@Pt, which are rich in undersaturated corner and edge sites, respectively. Reprinted with permission from [41]. Copyright 2018, American Chemical Society.

Understanding the ORR process is crucial for the development of effective catalysts. Despite disagreements surrounding the ORR process, it is generally accepted that the adsorption of intermediate species such as O and OH on metal catalysts may be the determining factor in the ORR performance of metal catalysts [11,38,39]. The ORR mechanism in acidic environments can be explained fundamentally as depicted in Figure 1a [40]. ORR is a multi-electron reaction comprising multi-step primitives containing numerous active intermediates, which can be categorized into two pathways: the dissociation pathway and the association mechanism. The dissociation pathway is the direct four-electron reaction between oxygen and hydrogen ions to produce water. In the association mechanism, oxygen preferentially combines with two electrons to form hydrogen peroxide, and some hydrogen peroxide then reacts with two electrons to form water. The ORR reaction commences with the adsorption of O_2_ on the surface of the catalyst, which controls the ORR’s electron mechanism. In determining the activity of catalysts in the ORR, adsorption is a crucial aspect. The three O adsorption modes on the catalyst’s surface are universally established. The Griffiths (single-site) and Bridge (dual-site) models are appropriate for 4e^−^ reactions, whereas the Pauling (single-site) model is appropriate for 2e^−^ reactions [41].

According to the widely accepted Sabatier principle in the field of catalysis, only the optimal adsorption strength of the catalyst for adsorbed reactants/intermediates is conducive to an efficient reaction [42,43]. Nørskov et al. [41] developed an approach based on the electronic structure to determine the stability of intermediates in an electrochemical reaction. Using density functional theory, they evaluated the catalytic ORR performance of Pt-based alloy on O and OH adsorption energies [44]. The relationship between ORR activity and oxygen adsorption energies was eventually demonstrated, as depicted in Figure 1b, by extending simulations to a range of noble metals [41]. As previously stated, Pt can be coupled with Ni, Co, Fe, and other elements to produce alloys, and all ORR intermediates can be linearly connected with the adsorption energy of OH (denoted Δ*E*_OH_). When metals weakly bind oxygen, the ORR rate is constrained by the dissociation of O_2_ or the transfer of electrons and protons to the adsorbed O_2_. Pt appears to be the most promising choice for the ORR, as well as potential ways of improving the ORR performance of metallic catalysts, as depicted by the volcano plot in Figure 1b [11].

Pt-based catalysts demonstrate the greatest performances for ORR of all currently available catalysts, rendering them almost irreplaceable. Generally, the surface shape, chemical composition, and electrolyte–electrode interfacing of electrocatalysts have the greatest influence on their activity, selectivity, and stability [45,46]. It is now well established that the reaction rate of the ORR on platinum single-crystal planes (Pt (hkl)) is structure-sensitive due to the adsorption of spectator species, such as OH, Br^−^, Cl^−^, etc., depending on surface structure [47]. Various studies on typical extended Pt-based surfaces, including Pt single-crystal metal electrodes, Pt monolayer, Pt skin, and near-surface alloy, have demonstrated that engineering a Pt surface is an effective way to optimize its catalytic properties for ORR [12,48,49,50]. Significantly, Stamenkovic et al. determined the ORR kinetics for Pt and Pt_3_Ni based on their surface structures and characteristics [31]. At 0.05 V, their findings revealed that the first atomic layer is composed entirely of Pt, whereas the second atomic layer is Ni-rich (52% Ni compared with 25% Ni in the bulk), and the third atomic layer is once again Pt-rich (87%). The adsorption of oxygenated species induces the contraction of Pt surface atoms, which is governed by the Ni-induced alteration of the Pt-skin electronic structure. The Pt_3_Ni (111) surface is 10-fold more active for the ORR than the similar Pt (111) surface and 90-fold more active than the current state-of-the-art Pt/C catalysts due to its unusual electronic structure and arrangement of surface atoms in the near-surface. The researchers subsequently established a fundamental electrocatalytic link between the experimentally determined surface electronic structure, i.e., the d-band center, and oxygen-reduction activity on Pt-bimetallic alloy surfaces [39]. The catalytic activity is determined with a balance between the adsorption energies of reactive intermediates and the surface coverage of the spectator (blocking) species [38,41]. The knowledge gained from the extended surfaces can be used to explain the activity pattern of the ORR on the existing Pt-based alloy nanocatalysts and also provide a fundamental basis for the rational design of nanocatalysts.

Inspired by the results of the extended surface, especially superior ORR activity on extended a Pt_3_Ni-skin (111) surface [31,39], recent studies have provided evidence of the high level of control and craftsmanship in the fabrication of well-defined nanocatalysts, which can yield high performance in both specific activity (SA) and mass activity (MA). Choi et al. [51] reported the synthesis of uniform 9 nm Pt-Ni octahedra using oleylamine and oleic acid as surfactants and W(CO)_6_ as structure-directing agents. They then removed the surfactants chemically adsorbed on the surface through acetic acid treatment. The obtained octahedra nanocatalysts with clean surfaces delivered an SA 51-fold higher than that of the state-of-the-art Pt/C catalyst for the ORR at 0.93 V, together with a contemporaneous record high MA of 3.3 A mg_Pt_^−1^ at 0.9 V. Alternatively, Zhang et al. [52] reported a scalable, surfactant-free, and low-cost solid-state chemistry method for the batch preparation of 2 g octahedral-carbon-supported Pt_1.5_Ni and demonstrated high ORR activity in the created catalyst. Huang et al. [50] modified a carbon-supported Pt_3_Ni octahedron with the transition metal Mo to create PtNiMo/C, which demonstrated excellent ORR performance with an SA of 10.3 mA cm^−2^ and an MA of 6.98 mA g_Pt_^−1^, which were 81- and 73-times that of the commercial Pt/C catalysts, respectively. According to recent studies, the near-surface composition of nanocatalysts and element isotropic distribution in the nanoparticle may account for the huge difference in the activity of Pt-Ni NPs, since the element segregation on the surface makes the structure of nanoalloys more elaborate relative to bulk alloys [53,54]. However, those results were just obtained from RDE tests; concrete evidence of activity and sufficient long-term stability under realistic fuel cell conditions is still lacking.

## 3. The Stability of Pt-Based Catalysts for ORR

**Figure 2:**
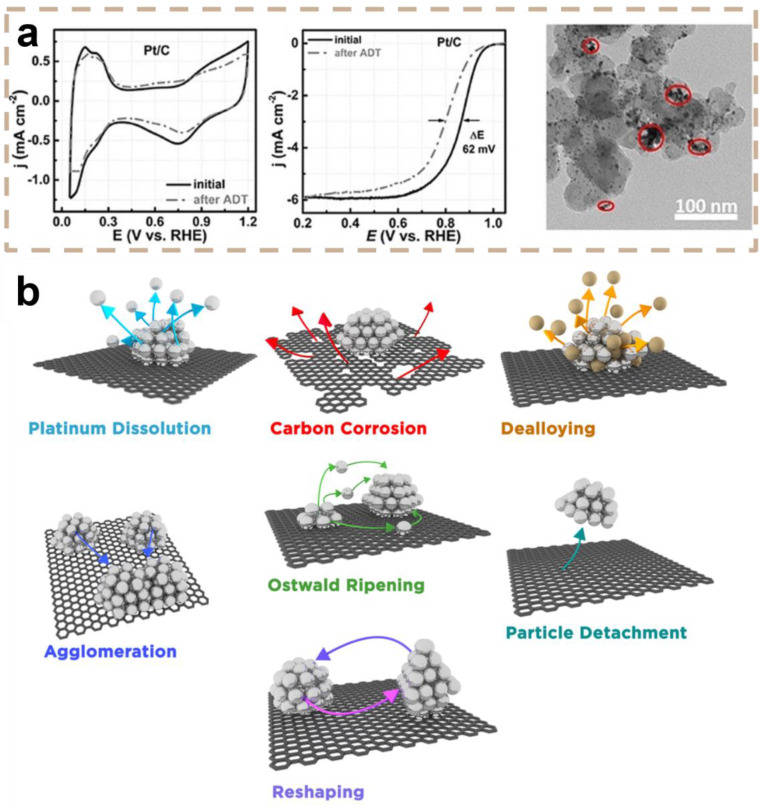
(**a**) Cyclic voltammetry curves and polarization curves of the commercial Pt/C before (solid) and after (dot) ADT, and TEM images of the commercial Pt/C after ADT. Red circles show the aggregation of Pt NPs. Reprinted with permission from [55]. Copyright 2021, John Wiley and Sons. (**b**) Schematic diagram of degradation mechanism of Pt-based catalyst. Arrows are the tracks of Pt species as they degrade. Reprinted with permission from [35]. Copyright 2016, American Chemical Society.

Considering that the prospect of further considerable improvement in the ORR activity of Pt-based catalysts is rather limited, long-term stability has become the most important challenge to be addressed. As shown in Figure 2a, the electrochemical surface area of commercial Pt/C catalysts became smaller, and the half-wave potential negatively shifted 62 mV after accelerated durability tests [55]. The transmission electron microscope (TEM) images showed a considerable aggregation of NPs and the corrosion of the carbon support, which may account for the degradation of the catalysts. Although the exact mechanism of the degradation of Pt-based catalysts still remains unclear, several widely accepted interrelated factors have been provided [6,35]: Pt dissolution, Ostwald ripening, particle detachment, the leaching of alloying metal, and the corrosion of carbon supports.

Platinum dissolution is an inevitable process (Figure 2b) [35]. Smaller Pt particles are more soluble as a result of their increased surface energy (the Gibbs–Thomson effect) [56,57]. In fuel cells, the dissolution of Pt from nanoparticle catalysts may result in a decrease or an increase in the Pt nanoparticle size (through Ostwald ripening) [58,59]. The dissolution of Pt at the cathode rises as the upper voltage limit is raised [60]. Although the precise process of Pt dissolution remains unknown, a link between Pt dissolution and the interchange of Pt-O sites at high potentials may account for the solubility of weakly bound Pt species [61].

Ostwald ripening is frequently followed by the dissolution of Pt; smaller particles dissolve while larger particles continue to develop, increasing the average particle size (Figure 2b) [35]. Currently, Ostwald ripening can be classified into two categories [6,59]: Three-dimensional Ostwald ripening is when Pt dissolves due to small NPs, the transit of dissolved Pt species, and the redeposition (reduction) of Pt species from a solution to big NPs. Two-dimensional Ostwald ripening is the alternative type. Bett et al. [59] hypothesized an alternative Ostwald ripening mechanism in which Pt would be transported by Pt molecular species on carbon supports.

Although the corrosion of typical carbon supports such as Vulcan is regarded as minimal at cell voltages below 0.8 V vs. RHE in low-temperature fuel cells, carbon corrosion and weight loss have been demonstrated to be substantial at voltages above 1.1 V vs. RHE [62,63]. Carbon corrosion can be observed under extreme potential settings for Pt-based catalysts supported by carbon (Figure 2b) [35]. Carbon corrosion can lead to shrinkage in the support structure or even the total removal of a component of aggregate, hence accelerating secondary degradation mechanisms such as particle aggregation and separation, and associated porosity loss can significantly restrict the mass transfer of reactants [64]. Consequently, the effectiveness of the electrochemical reactor is diminished. Various modified carbon supports, such as multiwalled carbon nanotubes [65], graphene [66,67], organized mesoporous or nanoporous carbon [37], and so on, have been developed to solve these issues. In several instances, the longevity of the catalyst was greater than that of conventional nanostructures; however, thorough knowledge of the effect of the support shape in retarding particular degradation pathways is still absent.

Carbon corrosion often induces the aggregation of Pt NPs and their separation from the carbon support [35,58]. The corrosion process depends on the cell voltage, the nature of interactions between Pt NPs and the carbon support, the degree of graphitization in the carbon support, and possibly other variables. The agglomeration of Pt particles will cause the particles to expand in size and decrease the utilization rate of platinum, hence reducing the ORR performance of the catalyst [55,58]. The agglomeration process happens because small particles have greater surface energy and tend to interact and produce larger particles with lower surface energy [68].

Particle detachment is the total loss of platinum particles that have separated from the support, resulting in a drop in the overall number of particles; it is an additional mechanism that has been observed using electron microscopy [58,68]. The detachment of particles originates from a weakening of the particle–support relationship and can, therefore, be triggered by the corrosion of the support [69]. As a result, agglomeration and separation are typically detected simultaneously following carbon corrosion.

Even in preleached alloys, the activity-boosting effect of the alloy metal is gradually lost during fuel cell operation, despite the fact that Pt-based alloy catalysts normally demonstrate significantly higher ORR catalytic activity [50,70]. In an acidic environment, the dissolving of the non-noble metal surface atoms is thermodynamically favorable at the potentials to which the ORR catalysts are exposed, and the alloy material in the core of Pt alloy catalysts with rich Pt shells is also vulnerable to dissolution. PEMFCs can be severely damaged by the interchange of proton sites with metal cations in the ionomer [27,71]. Consequently, the resistance of the ionomer in the membrane and catalyst layer rises, the oxygen diffusion rate in the ionomer falls, and peroxy free radicals damage the proton exchange membrane. Furthermore, dissolved metal ions may pass through the membrane and deposit on the anode of the fuel cell [72].

## 4. Strategies for Stability Enhancement of Pt-Based Catalysts

### 4.1. Intermetallic Compound

The long-term performance of electrocatalysts depends upon the atomic-level chemical and structural stability of the active sites. As mentioned in the last section, when Pt is alloyed with a transition metal (M = Co, Fe, Ni, Cu, etc.), Pt-based alloy catalysts typically exhibit greatly enhanced ORR activity [73,74]. However, in Pt-based alloy electrocatalysts, non-noble transition metal elements can be selectively leached from the NPs, resulting in the structural deformation of the highly active sites and a decline in catalyst performance, which makes it difficult to transfer its enticingly high performance into a realistic membrane electrode assembly (MEA) for activity and stability. Due to their intrinsic thermodynamic instability, alloying metal atoms tend to dissolve into the electrolyte, especially during operation, causing not only a major decrease in the electrocatalyst’s activity but also severe damage to other functional components, such as the Nafion membrane [17,27,71,75]. To improve the electrochemical stability of Pt and M alloys, it has been demonstrated that catalysts with core–shell nanostructures can be fabricated in which the nuclei of M-rich alloy NPs are encased in Pt shells with multiatomic layer thickness, thereby decreasing the dissolution pathway of M atoms [48,76,77].

**Figure 3:**
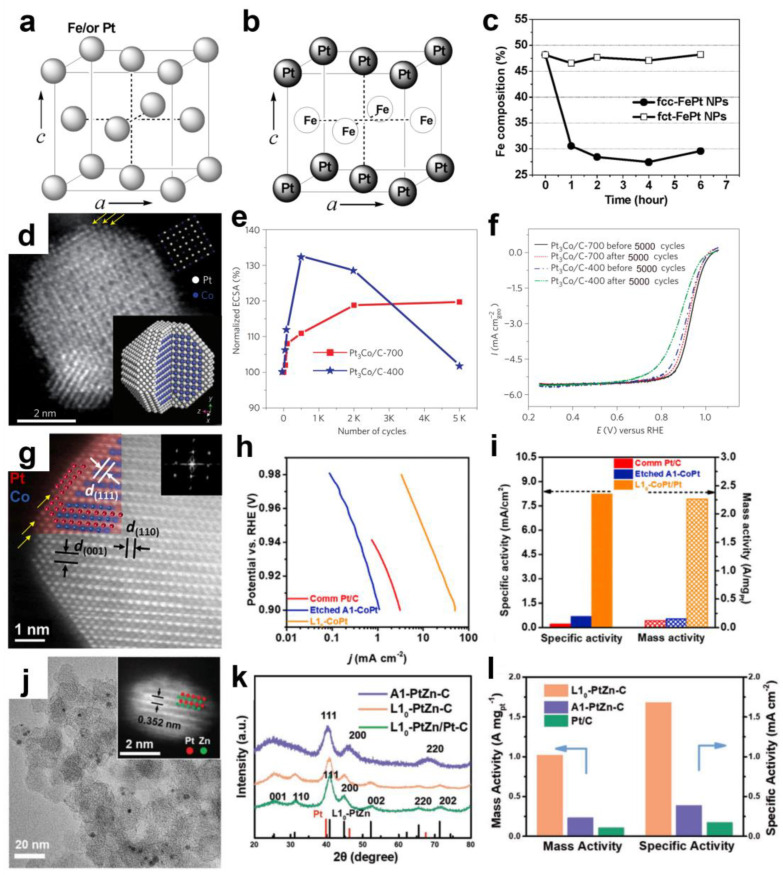
Schematic illustration of (**a**) chemically disordered fcc-FePt and (**b**) chemically ordered fct-FePt. (**c**) The composition of fcc- and fct-FePt NPs in 0.5 M H_2_SO_4_ solution varies with time. Reprinted with permission from [32]. Copyright 2010, American Chemical Society. (**d**) Atomic-resolution annular dark-field (ADF) scanning transmission electron microscopy (STEM) image of Pt_3_Co/C-700 after RichardsonLucy deconvolution, with yellow arrows indicating the Pt-rich shell. The upper inset shows the projected unit cell along the [001] axis, and the lower inset shows the idealized atomic structure of the Pt_3_Co core–shell nanoparticle. The white and blue spheres in the lower inset represent Pt and Co atoms, respectively. (**e**) Electrochemically active surface area (ECSA) as a function of the number of cyclic voltammetry cycles for Pt_3_Co/C-400 and Pt_3_Co/C-700 catalysts. (**f**) Comparative ORR activities of Pt_3_Co/C-400 and Pt_3_Co/C-700 catalysts before and after 5000 potential cycles. Reprinted with permission from [48]. Copyright 2012, Springer Nature. (**g**) Enlarged sections indicated by dashed squares show the 2–3 atomic layers of the Pt shell (indicated by yellow arrows) and the L1_0_-CoPt core; the red and blue spheres in (**g**) represent Pt and Co atoms, respectively. (**h**) Tafel plots of comm Pt/C, etched A1-CoPt, and L1_0_-CoPt/Pt. (**i**) SA and MA of comm Pt/C, etched A1-CoPt, and L1_0_-CoPt/Pt measured at 0.9 V vs. RHE. Reprinted with permission from [80]. Copyright 2019, Elsevier. (**j**) TEM image of L1_0_-PtZn-C after thermal annealing at 600 °C. The inset is a high-angle annular dark-field (HAADF) scanning transmission electron microscopy (STEM) image of a representative L1_0_-PtZn NP. (**k**) X-ray diffraction (XRD) patterns of A1-PtZn-C, L1_0_-PtZn-C, and L1_0_-PtZn/Pt-C. (**l**) MA and SA of L1_0_-PtZn-C, A1-PtZn-C, and Pt/C at 0.9 V vs. RHE. Reprinted with permission from [86]. Copyright 2020, John Wiley and Sons.

To prevent the leaching of transition metal atoms, among the several core–shell alloy electrocatalysts, very structurally stable ordered Pt alloys can be produced. Due to the strong Pt-M contact and stronger Pt-M bonding, highly ordered alloys or intermetallic compounds control the local atomic structure of Pt, resulting in more advanced intrinsic characteristics [76,78,79]. In 2013, Wang et al. [48] developed a unique class of Pt-Co nanocatalysts comprising an ordered Pt_3_Co intermetallic compound core and two to three atomic layers of platinum shells (Figure 3d). Compared with disordered Pt_3_Co alloy NPs and Pt/C, the MA and SA of these ordered Pt_3_Co intermetallic compounds increased by more than 200 and 300%, respectively. Figure 3e,f demonstrate that even after 5000 potential cycles, the activity loss of the ordered alloy catalyst is negligible, and the ordered core–shell structure is nearly intact. The Pt-rich shell and stable intermetallic Pt_3_Co core arrangement are responsible for the high activity and stability.

Sun and colleagues [80] report a class of atomically ordered, strongly ferromagnetic CoPt NPs as outstanding ORR electrocatalysts, surpassing the technical targets established by the United States Department of Energy in the half-cell rotating disk electrode (RDE) and full-cell MEA settings. They suggest that the majority of documented failures to stable M in improved PtM electrocatalysts are mostly attributable to their cubic phase in solid solutions or intermetallic compounds [32]. They hypothesize that the tetragonal phase can produce a stronger coupling link between M and Pt along the crystal c direction than the cubic phase, thus providing the advantageous M with increased resistance to electrochemical and chemical etching (the cubic phase is Figure 3a; the tetragonal phase is Figure 3b). Sun and colleagues [32] introduced the idea in 2010, demonstrating that L1_0_ FePt NPs are significantly more stable for Fe than their face-centered cubic (fcc) FePt counterpart (Figure 3c), with increased ORR activity. Since then, the L1_0_ FePt system has been vastly upgraded and even implemented in a real MEA.

The L1_0_ CoPt catalyst was then created by heating 9 nm disordered solid solution CoPt NPs generated by wet chemistry [80]. Figure 3g depicts a core–shell structure composed of tetragonal CoPt nuclei and pure Pt shells with an atomic thickness (denoted as L1_0_ CoPt/Pt). Figure 3h,i demonstrate that the SA and MA of this structure are 38- and 19-times greater than the Pt/C, respectively. Impressively, more than 80% of the original MA and the majority of the advantageous Co may be kept over 30,000 extraordinarily long potential cycles, emphasizing once again the significance of the tetra phase in stabilizing inexpensive transition metals in catalysts.

Despite the promise of ordered alloys, controlling the particle size of the metal is very challenging since most intermetallic compounds are generated by annealing at elevated temperatures. As a result, NPs tend to agglomerate excessively, reducing the electrochemistry area and Pt utilization. Typically, metal oxide [81,82] or carbon coatings [83,84,85] are utilized to prevent particle agglomeration or sintering, and these coatings might impact the movement of NPs, leading to the creation of partially ordered structures. ZnO has been employed more recently as a bifunctional precursor to offering a diffusion of Zn atoms to the Pt lattice, where it also acts as a physical barrier to prevent the sintering of particles during heat treatment (Figure 3j–l) [86]. Contributing to high ORR activity is the strong ion/metal interaction between Zn and Pt, with stable Pt lattice parameters and a unique biaxial strain, with tension along the (110) direction and compression along the (101) and (011) directions.

### 4.2. Surface Modification

The ORR electrocatalysis of Pt-based alloy catalysts is sensitive to the surface’s local electronic structure and atomic coordination environment [87]. Surface engineering methodologies have been frequently employed to change binary or ternary alloy NPs, and certain surface-doped NPs have demonstrated enhanced catalytic activity [7,88].

**Figure 4:**
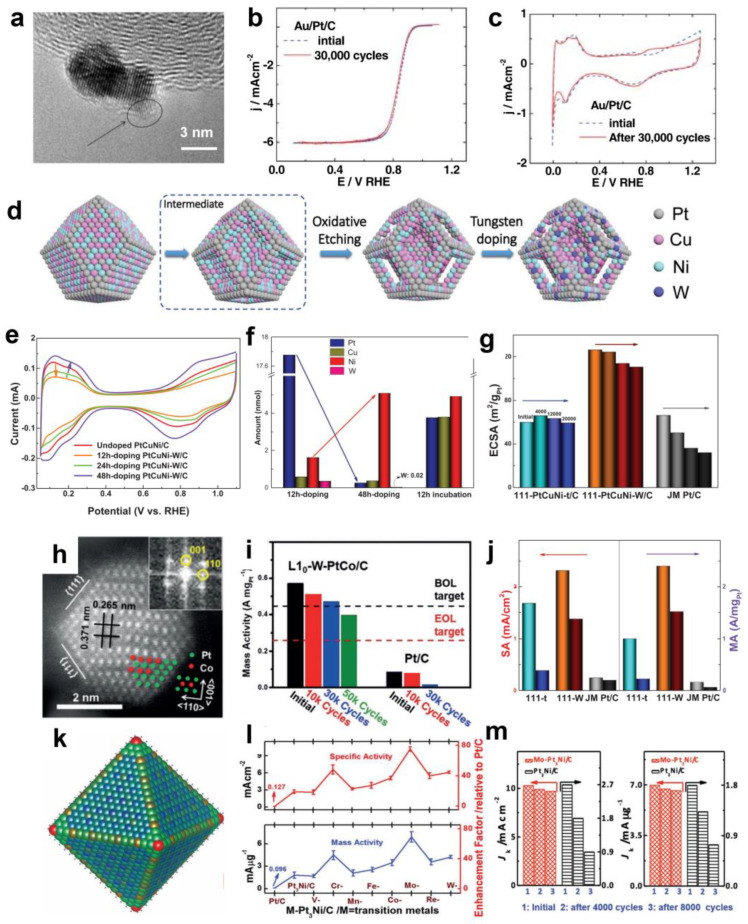
(**a**) High-resolution transmission electron microscopy (HRTEM) image of an Au/Pt/C catalyst made via the displacement of a Cu monolayer with Au. A different structure in the areas indicated by the arrows is ascribed to the Au clusters. (**b**) Polarization curves for the ORR on Au/Pt/C catalysts on an RDE, before and after potential cycles. (**c**) CV curves for Au/Pt/C catalysts before and after cycles. Reprinted with permission from [33]. Copyright 2007, Springer Nature. (**d**) Schematic illustration of the morphology evolution in the acid treatment and W doping process. (**e**) CV curves of 111-PtCuNi-W/C with W doping. (**f**) Detected amounts of Pt, Cu, Ni, and W in the supernatants of reaction solution with various W doping or incubation (without W precursor) times. (**g**) Evolutions of calculated ECSA for 111-PtCuNi-t/C, 111-PtCuNi-W/C, and JM Pt/C. Reprinted with permission from [91]. Copyright 2019, John Wiley and Sons. (**h**) HAADF-STEM image of a L1_0_-W-PtCo NP. Inset: corresponding FFT pattern. (**i**) MA of L1_0_-W-PtCo/C and Pt/C at 0.9 V_iR-free_ before and after voltage cycles at 80 °C. Reprinted with permission from [92]. Copyright 2019, John Wiley and Sons. (**j**) Evolutions of SA and MA of 111-PtCuNi-t/C, 111-PtCuNi-W/C, and JM Pt/C. (**k**) The average site occupancies of the second layer of the Mo_73_Ni_1143_Pt_3357_ NC at 170 °C as determined using a Monte Carlo simulation. The red, green, and blue spheres in the lower inset represent Mo, Ni, and Pt atoms, respectively. (**l**) The SA and MA at 0.9 V vs. RHE for these transition-metal-doped Pt_3_Ni/C catalysts. (**m**) The changes in the SA and MA of the octahedral Mo-Pt_3_Ni/C catalyst and octahedral Pt_3_Ni/C catalyst before and after 4000 and after 8000 potential cycles. Reprinted with permission from [50]. Copyright 2015, Springer Nature.

The use of gold as a doping agent is of great interest to researchers. Adzic et al. [33] discovered in 2007 that altering a tiny number of gold clusters surrounding Pt NPs can significantly enhance the stability of Pt NPs, even at 0.6–1.1 V vs. RHE for 30,000 cycles, as shown in Figure 4a–c. The introduction of gold clusters can enhance the oxidation potential of Pt, hence lowering Pt dissolution and migration and enhancing the material’s stability. Au atoms may spread to low coordination sites such as edges and steps, preventing these sites from interacting with the solution and thus decreasing the rate of Pt dissolution. Numerous studies have since demonstrated that Au doping can enhance the stability of catalysts such as Au-PtNi NPs [54,89] and Au-PtCu NPs [90]. Lu et al. [90] demonstrated that the stability of PtCu/C can be greatly improved by adding trace Au (Au/Pt = 0.0005). After 10,000 potential cycles, the MA loss of PtCuAu_0.0005_/C was only 8%, significantly less than that of PtCu/C and Pt/C. In sum, Au doping has great potential to enhance the stability of catalysts.

W doping is also an excellent way to increase endurance. Li et al. [91] developed a highly active PtCu_x_Ni ternary alloy catalyst with a porous structure utilizing an acid treatment technique and introducing W atoms as a surface doping agent. The schematic diagram is depicted in Figure 4d. Intriguingly, surface W doping can promote atomic rebuilding on the catalyst surface, primarily involving Pt and Ni atoms. Using inductively coupled plasma emission spectroscopy (ICP-OES) to evaluate the concentration of metal compounds in the supernatant, it was discovered that the Pt concentration in the supernatant increases significantly to 17.75 nmol after 12 h of W doping. The concentration of W continued to decrease, but the concentration of Ni exhibited a reverse trend (Figure 4f). Simultaneously, this can drastically alter the electrochemical surface area and increase the MA somewhat more than the activity of the mass (Figure 4e–g). Additionally, W surface doping increases the stability of the PtCu_x_Ni alloy catalyst. The ECSA did not reduce considerably for undoped catalysts after 20,000 accelerated stability cycles, although the SA and MA decreased dramatically by 77.3% and 78.8%, respectively. Although the ECSA reduced by 12.5% following W doping, the SA and MA of the catalyst decreased by 37.7% and 34.5%, respectively (Figure 4j). Therefore, the addition of W atoms to the surface of PtCu_x_Ni ternary alloy can efficiently regulate its activity and durability.

In Figure 4h, Li and his colleagues [92] produced a 3 nm ordered L1_0_ PtCo nanoparticle catalyst and then attempted to introduce modest amounts (5–7 atom%) of metals (i.e., W, Ga, and Zn) to the PtCo lattice. The L1_0_-W-PtCo/C catalyst demonstrated remarkable ORR activity and stability, as well as outstanding performance in PEMFC testing (Figure 4i). The initial MA of the L1_0_-W-PtCo/C catalyst was 0.57 A mg_Pt_^−1^, and after 30,000 cycles, only 17.5% of the MA was lost. In addition, extended X-ray absorption fine structure (EXAFS) analysis and density functional theory (DFT) calculations demonstrate the crucial role of W doping in enhancing ORR performance. They suggest that W doping can modify the Pt-Pt distance and the surface Pt strain and optimize the surface energy to stabilize the nanoparticle structure.

Huang and colleagues [50] completed one of the most representative studies on surface engineering when they investigated transition-metal-doped Pt_3_Ni NPs. Mo doping catalysts have the highest ORR activity across all types of dopants, including V, Cr, Mn, Fe, Co, Mo, Re, and W (Figure 4l). The MA of Mo-Pt_3_Ni is 6.98 A mg_Pt_^−1^, and its SA is 10.3 mA cm^−2^. In addition, after 8000 durability tests, MA barely degrades by 5.5%, as demonstrated in Figure 4m. Theoretical DFT calculations indicate that the addition of Mo can create strong Mo-Pt and Mo-Ni bonds, which can stabilize Pt and Ni and reduce elemental dissolution, hence enhancing catalytic stability. In a recent paper, researchers [93] from the same research group employed in situ synchrotron radiation and molecular dynamics modeling to determine that the Mo element predominantly occurs in an oxidation state and preferentially occupies the boundary, the vertex, and other poor coordination locations (the form is shown in Figure 4k). The introduction of Mo can limit the migration of nearby Pt atoms and stabilize the octahedral structure of nanocrystals. The Ni within the nanocrystalline can be protected by the Pt shell on the surface to prevent Ni etching and loss, thus protecting the Pt_3_Ni (111) crystal surface and enhancing the catalyst’s stability. In addition to W doping and Mo doping, Rh doping [94,95] and Ru doping [96] can also significantly enhance the catalyst’s stability.

### 4.3. Self-Supporting Material

**Figure 5:**
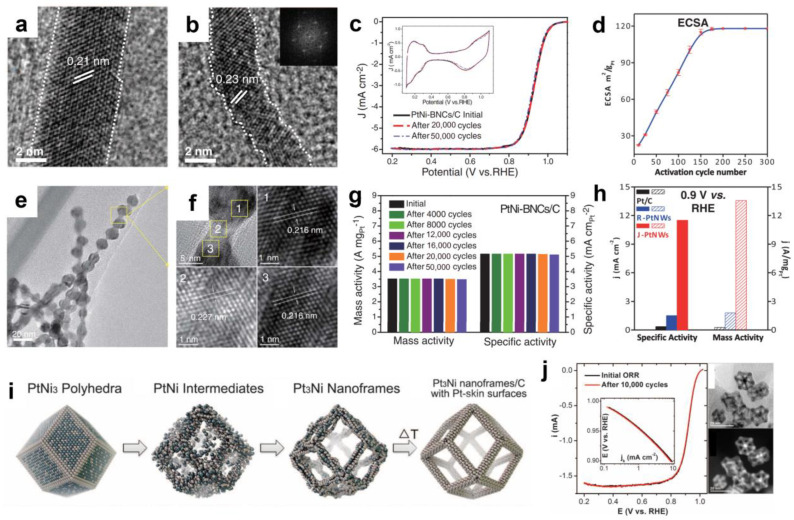
(**a**,**b**) HRTEM images of PtNi alloy nanowires and J-PtNWs supported on carbon. (**c**) ORR polarization curves and (inset) corresponding CV variations. (**d**) The evolution of ECSA with an increasing number of CV cycles shows that 160 cycles are sufficient to construct the J-PtNW and reach a stable ECSA. (**e**) TEM images of PtNi-BNSs. (**f**) Enlarged TEM image of the area indicated in (**e**) and the corresponding HRTEM images of the areas marked by yellow squares. (**g**) Mass and SA evolutions for PtNi-BNCs/C before and after the durability test for various potential-scanning cycles. Reprinted with permission from [98]. Copyright 2016, Springer Nature. (**h**) Comparison of the SA and MA of the J-PtNWs, R-PtNWs, and Pt/C catalyst at 0.9 V vs. RHE. Reprinted with permission from [97]. Copyright 2019, Springer Nature. (**i**) Schematic illustrations of the samples obtained at four representative stages during the evolution process from polyhedra to nanoframes. (**j**) ORR polarization curves and (inset) corresponding Tafel plots of Pt_3_Ni frames before and after 10,000 potential cycles between 0.6 and 1.0 V vs. RHE. The images on the right are bright-field STEM images and dark-field STEM images of Pt_3_Ni nanoframes/C after cycles. Reprinted with permission from [49]. Copyright 2014, Springer Nature.

In comparison with other anisotropic nanostructures, one-dimensional nanowires (NWs) have attracted the most interest due to their high aspect ratios and specific surface areas, which can increase Pt utilization efficiency. In addition, the asymmetry of the nanowire structure can alleviate the durability issue by efficiently inhibiting the degradation modes of the catalyst, such as dissolution, Ostwald ripening, and aggregation. In Pt nanoalloys with NW structures, outstanding ORR performance has been easily obtained due to the aforementioned benefits. Pt-Ni-bunched nanocages with a Pt skin surface (Figure 5e,f) exhibited 17- and 14-times the MA (3.52 A mg_Pt_^−1^) and SA (5.16 mA cm^−2^) of commercial Pt/C catalysts, respectively [97]. Due to the substantial amount of activation energy lost by surface Pt atoms, the Pt surface structure hinders the dissolution of surface Pt and the further leaching of Ni species, even in the presence of oxygen adsorbent. They also demonstrated low activity attenuation after 50,000 cycles (Figure 5c,g), and the PtNi nanocages demonstrated a high level of service in H_2_–air fuel cell installations that were run at 0.6 V vs. RHE for 180 h.

In 2016, ultrafine jagged Pt NWs were produced by electrochemically dealloying PtNi alloy NWs [98]. The dealloying process removes Ni atoms and rearranges the remaining Pt atoms into a zigzag pattern, resulting in a surface configuration that is highly strained and poorly coordinated (Figure 5a,b). Due to this distinctive surface structure, J-Pt NWs exhibited a good ECSA (Figure 5d,h), and jagged nanowires exhibited a specific ORR activity of 11.5 mA cm^−2^ and an MA of 13.6 A mg_Pt_^−1^ at 0.9 V vs. RHE, respectively.

In addition to one-dimensional nanowires, several interconnected and highly open structures have been demonstrated to be adequately reactive and less susceptible to sintering, dissolution, and separation as a result of their increased size and enhanced contact. The development of nanoframes is crucial for enhancing the performance of electrocatalysis in ORR [5,49,99]. Chen et al. [49] showed the erosion-induced synthesis of Pt_3_Ni nanoframe nanostructures from PtNi_3_ polyhedra in 2014 (Figure 5i). In a typical experiment, a PtNi_3_ polyhedron with a homogeneous rhombic dodecahedral structure was created and eventually etched into extremely open hollow nanoframes, resulting in a change from PtNi_3_ to PtNi to Pt_3_Ni in the bulk composition. A Pt_3_Ni NFs/C catalyst with a Pt skin surface was produced by distributing Pt_3_Ni NFs on a carbon support and then subjecting the material to heat treatment. The resultant Pt_3_Ni nanoframe was subsequently utilized for the electrocatalysis of ORR, exhibiting greatly improved activity at 0.95 V vs. RHE and being stable even after 10,000 cycles (Figure 5j). The shift in the electronic structure of the Pt layer on top of the Pt_3_Ni alloy is responsible for the weak oxygen binding strength, which contributes to enhanced activity and stability.

### 4.4. Support Enhancement

In addition to the building of stable intermetallic compounds, surface modification to change the stability of NPs, and the preparation of anisotropic self-supporting materials, the stability of Pt-based catalysts can be enhanced via the use of modified carbon supports or carbon coating [29,37,100,101].

**Figure 6:**
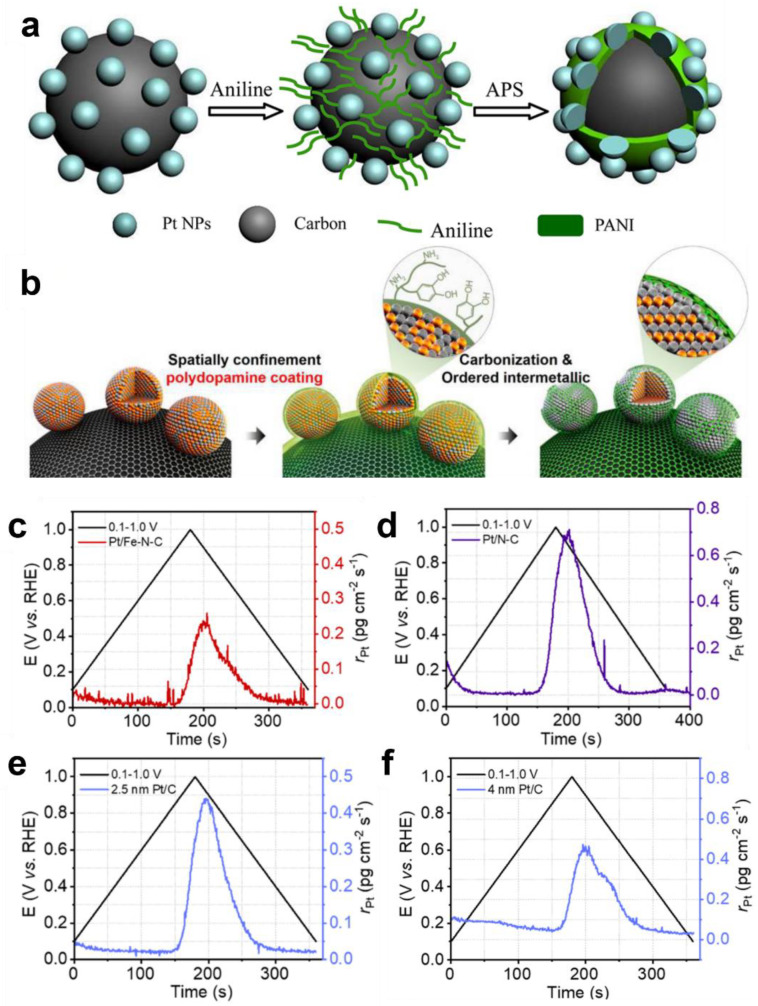
(**a**) Configuration of the Pt/C@PANI catalyst. Reprinted with permission from [102]. Copyright 2012, American Chemical Society. (**b**) Schematic synthesis diagram of carbon-supported and N-doped carbon-coated ordered fct-PtFe NPs. Dissolution rates of Pt for Pt/Fe-N-C. Reprinted with permission from [85]. Copyright 2015, American Chemical Society. (**c**), Pt/N-C (**d**), 2.5 nm Pt/C (**e**), and 4 nm Pt/C (**f**) catalysts were recorded during a single potential cycle in 0.1 M KOH at 5 mV s^−1^. Reprinted with permission from [37]. Copyright 2022, American Chemical Society.

Due to the high surface energy of Pt NPs supported by carbon black, the interaction between the catalyst and the support is rather weak. During fuel cell operation, particularly during the start–stop stage, the potential fluctuates greatly, and the instantaneous potential can reach a very high value. It is easy for Pt particles to disintegrate, migrate, and redeposit on the surface of the support, a phenomenon known as Oswald ripening [35]. Consequently, the active surface area of the catalyst is diminished, and the performance of the fuel cell steadily declines. Several experiments have been conducted to create Pt/C@PANI core–shell catalysts by altering Pt/C with conductive polyaniline and utilizing π-π interactions between polyaniline and carbon supports to selectively load polyaniline onto carbon rather than Pt surfaces [102]. The addition of polyaniline enhanced the conductivity and proton conductivity of the catalytic system, while the Pt active site remained unblocked, and the activity of the catalyst was effectively sustained (Figure 6a). Moreover, polyaniline coating considerably increased the catalyst’s stability. Single-cell test results indicate that after 5000 cycles, the corresponding current density of 0.6 V vs. RHE for a battery with Pt/C@PANI as the cathode catalyst decreases slowly from 1.17 to 0.89 A cm^−2^, whereas for the battery assembled with commercial Pt/C, the corresponding current density decreases rapidly from 1.14 to 0.16 A cm^−2^. There are two explanations for polyaniline’s increased stability: First, the electron transport between Pt and polyaniline makes the oxidation of Pt NPs more difficult. Second, the coating of polyaniline on the carbon layer limits the corrosion of the carbon support to some extent. Although the catalyst’s stability has been enhanced, it still has some limits. When pH exceeds four, polyaniline acts as an insulator, resulting in low catalyst conductivity.

In addition to coating Pt NPs with polyaniline to avoid their sintering, the surface of the NPs can be coated with oxides [82,103] and other heat-stable compounds [83,84,85,102] to prevent their aggregation. For instance, Chung et al. coated PtFe NPs with polydopamine [85]. During the annealing procedure, dopamine was carbonized in situ to produce a N-doped carbon protective layer that shielded PtFe NPs. The PtFe NPs themselves underwent a phase shift to create L1_0_ PtFe NPs at the same time (Figure 6b). In comparison with disordered PtFe NPs and commercial Pt/C, L1_0_ PtFe NPs demonstrated superior ORR activity with an MA of 1.6 A mg_Pt_^−1^. The catalyst was subsequently utilized in fuel cells. Due to the protection provided by the carbon layer and the unique ordered structure, the power density of the catalyst was reduced by only 3.4% over a 100 h test of stability, demonstrating outstanding stability. The power density of commercial platinum carbon was reduced by 27% under the same conditions.

Improving the interaction between the catalyst and support is essential for enhancing the stability of Pt-based catalysts. Using modified carbon as the support for a Pt-based catalyst, such as nitrogen-doped carbon (N-C) [83,85], metal nitrogen–carbon (Me-N-C) [12,37,101], etc., the stability of the Pt-based catalyst is strengthened by strong metal support contact. After 5000 cycles on RDE, intermetallic Pt-Fe alloy particles scattered at Fe-N-C sites demonstrated low activity loss, as reported by the Liu group [101]. The study indicates that Fe-N-C may support Pt NPs better than conventional carbon with a high surface area. Shao et al. [37] discovered that the electrochemical surface area retention rate of Pt/Fe-N-C in acidic and alkaline environments is much superior to that of commercial Pt/C. Using in situ inductively coupled plasma mass spectrometry, they demonstrated that the dissolution rate of Pt/Fe-N-C during cycling is three times lower than that of Pt/C. (Figure 6c–e). In addition, it is known from the scientific literature that larger Pt NPs are more stable than smaller ones and that the corresponding Pt atom leaching degree is lower. Through comparison, they ruled out the effect of nanoparticle size on the leaching of Pt atoms, and the stabilizing effect of Fe-N-C on Pt NPs is independent of nanoparticle size (Figure 6f). Calculations based on the density functional theory reveal further that Fe-N-C substrates can provide strong and stable support for Pt NPs and reduce the development of oxides by modifying the electronic structure. The greatly increased Pt/Fe-N-C stability may be attributable to the strong metal–matrix interaction, along with the low metal dissolution rate and extremely stable support.

## 5. Summary and Prospect

At present, the design and development of stable and efficient Pt-based PEMFC cathode catalysts have attracted considerable interest. Researchers have been able to precisely design and construct nanocrystalline catalysts with adjustable sizes, shapes, and structures, thereby significantly enhancing the oxygen reduction activity of catalysts and reaching exceptional results. In contrast to activity, enhancing the long-term performance and stability of Pt-based ORR catalysts is urgently needed. This review has provided a survey of the recent developments in the stabilization of Pt-based catalysts in reactions related to fuel cells, and we have tried to present an up-to-date overview of this rapidly growing field. Several ways have been proposed to improve the stability of the catalyst, including doping with transition metals, ordered alloy building, support reinforcement, etc., and some success has been obtained. In contrast with the activity, however, the study of catalyst stability offers a great deal of room for growth.

In the foreseeable future, more endeavors should be focused on the following directions: (1) The development of new and upgraded ORR catalysts is an important option for the sustainable development of fuel cells. In addition, it is necessary to create a simple, surfactant-free, and environmentally friendly process for the mass manufacturing of industrial durability catalysts. (2) Due to the huge discrepancy in the evaluation criteria of RDE and MEA, highly active and durable catalysts cannot maintain similar performance to that obtained on RDE at the MEA level. As a result of uncontrolled catalyst layer thickness, large mass transfer resistance, and limited accessibility to active sites, the complicated membrane electrode assembly processes have limited performance. The catalyst’s activity and stability in the fuel cell must be tested as thoroughly as is feasible instead of only with RDE tests since the operating temperature and membrane electrode structure of fuel cells are substantially distinct from those of the half-cell test. (3) Based on the results of in situ/operando characterization techniques in degradation mechanism analysis, using advanced microscopic analysis instruments will find out the actual deactivation mechanisms, such as dissolution, oxidation, corrosion, and aggregation, which will provide new ways to rationally design highly active and durable ORR electrocatalysts. For a comprehensive understanding of the mechanism of catalyst stability attenuation and to facilitate the development of theoretical models, considerable efforts should focus on developing in situ/operando characterization and testing instruments that can track structural changes in real-time. (4) For the rational design of stable catalysts, it is urgent to develop plausible and universal models and identify appropriate stability descriptors. In summary, the promotion of intriguing PEMFCs to the public needs constant advances in the design and production of more efficient catalysts, as well as the resolution of a number of complex scientific and technical problems.

## Data Availability

The data presented in this study are available upon request.
